# Dissociation between the processing of humorous and monetary rewards in the ‘motivation’ and ‘hedonic’ brains

**DOI:** 10.1038/s41598-018-33623-4

**Published:** 2018-10-18

**Authors:** Yu-Chen Chan, Wei-Chin Hsu, Tai-Li Chou

**Affiliations:** 1 Department of Educational Psychology and Counseling, National Tsing Hua University, Hsinchu, Taiwan; 2 Research Center for Education and Mind Sciences, NTHU, Hsinchu, Taiwan; 3 Graduate Institute of Applied Science and Technology, National Taiwan University of Science and Technology, Taipei, Taiwan; 4 Department of Psychology, National Taiwan University, Taipei, Taiwan

## Abstract

Humor elicits feelings of amusement and can be thought of as a social reward. We identified distinct mesolimbic reward system (MRS) processing patterns for monetary and humorous rewards. During both the reward anticipation and outcome phases, the nucleus accumbens (NAc) and anterior cingulate cortex (ACC) were active in response to monetary cues and monetary gains, while the amygdala and midbrain showed a differential response to humorous rewards, apparently driven by the hedonic enjoyment and appreciation of humor consumption. Psychophysiological interaction analysis (PPI) further demonstrated the functional coupling of the *amygdala-midbrain* circuit in response to humorous gains during the reward outcome phase, while neural signaling was observed in the *NAc-ACC* circuit during both the reward anticipation and outcome phases in response to monetary rewards. This is consistent with a view in which the NAc plays a key role in the ‘motivation brain’, and the amygdala in the ‘hedonic brain’. The findings further suggest that the neural mechanisms underlying reward consumption are more modality-specific than those underlying reward anticipation. Our study contributes to a growing understanding of neural responses to social rewards and represent an important first step toward understanding the neural processing of humor as one significant type of social reward.

## Introduction

Affective neuroscience has recently emerged as an exciting discipline evaluating the neural correlates of motivation, affect, and emotion. As part of this, a growing body of research has focused on reward processing. Reward processing involves a reward anticipation phase and a reward outcome phase, and both phases involve motivational, learning, and affective components. The reward anticipation phase is characterized by wanting, the experience of incentive salience that triggers a motivational impulse to obtain a reward, while the reward outcome phase is characterized by liking, the hedonic enjoyment experienced upon consuming the reward.

Animal studies have investigated the distinct neural substrates associated with ‘wanting’ and ‘liking’^1–3^. Functional magnetic resonance imaging (fMRI) studies investigating reward processing based on blood oxygen level-dependent (BOLD) have detected differences between the delivery of reward cues that predict the occurrence of a reward (i.e., signal reward anticipation) and the reception of rewards (i.e., signal reward feedback) in humans^4^. Recent studies have examined the neural circuitry involved in distinct phases of reward processing, including anticipation of feedback, response to outcomes, and the combination of anticipation and response to outcomes^4,5^.

Previous reward processing studies have primarily relied on a conventional monetary incentive delay (MID) task^6–8^ or a modified MID task^9,10^. However, there has been a growing interest in identifying the neural substrates of appetite and consumption using both MID and social incentive delay (SID) tasks^11,12^. In this study, we pursue this direction by focusing on humor as a type of social reward. Humor often occurs in social interactions, plays a key role in social incentives and can elicit feelings of amusement^13^.

Monetary rewards elicit pleasure and humorous rewards elicit feelings of amusement during the hedonic consumption phase. Both of these experiences are generated by MRS circuits but whether and how the neural substrates underlying these experiences differ or are shared between monetary and humorous reward types remain unclear. Although at least one behavioral study has investigated the effort required to obtain humorous versus non-humorous cartoon rewards^14^, we are aware of no neuroimaging studies that have investigated the motivational and hedonic affective processing that occurs in response to humor. The present study employed a humorous incentive delay (HID) task along with a MID task to investigate whether humorous and monetary rewards recruit different regions of the MRS.

Several previous studies have shown that the ventral striatum, particularly the NAc, plays a key role during the anticipation of monetary rewards with an expected positive value^6–8^. The NAc is generally known to be associated with the MRS during reward prediction^5,6,15^ and dopamine release in the NAc during reward anticipation is more robust than that during reward consumption^1,6–8^.

The anterior cingulate cortex (ACC) plays a role in reward anticipation, target detection, and reward value encoding^16^. The ACC appears to be more involved in efforts directed toward task goals, cognitive control, and emotion-attention interactions during reward anticipation than during reward consumption^8^. The ACC is also more consistently activated during the anticipation phase than during the outcome phase^4^. However, whether the neural correlates of anticipation motivation in the NAc and ACC regions differ in response to different reward types, such as monetary and humorous rewards, is unclear.

Humor involves both comprehension (‘getting’ a joke) and appreciation (‘enjoying’ a joke)^17–26^. Numerous fMRI studies investigating humor appreciation have suggested that the feeling of amusement is associated with the MRS, including the amygdala, midbrain, NAc and ACC^13,18–24^. The ventral system, which comprises the amygdala, midbrain, NAc, and ventral ACC (vACC), is associated with the generation of the positive experiences of amusement (a bottom-up emotional response), while the dorsal system, which comprises the dorsal ACC (dACC), is associated with the voluntary, top-down, cognitive control of emotion^13,27–29^.

The primary purposes of the present study were to examine whether humorous and monetary rewards recruit different regions of the MRS during the anticipation phase and during the outcome phase of reward processing. Accordingly, we sought to identify an interaction between reward type and phase in the neural mechanisms underlying reward processing. Three reward types, monetary (M), humorous (H), and no reward (N), were used. Reward processing includes both the anticipation (A) and outcome (O) phases. The present study thus included six conditions: MA, MO, HA, HO, NA, and NO.

Neural activity in the MRS is related to dopamine release. A priori ROIs, including the NAc, ACC, amygdala, and midbrain, were used for the analysis of neural activity in this study. In addition, we employed a psychophysiological interaction analysis (PPI) to examine the functional connectivity of the NAc and amygdala during reward-related activation. Based on previous MID studies^6–8,12^, we predicted that the activation of the NAc and ACC would be greater during the anticipation of a monetary reward than during the anticipation of a humorous reward. We also predicted that the activation of the NAc and ACC during the reward anticipation phase would be greater than during the reward outcome phase in response to a monetary reward type. Based on previous studies using social incentives and studies of humor processing^12,13,18–24^, we predicted that the activation of the amygdala and midbrain upon receiving a humorous reward would be greater than upon receiving a monetary reward. We further predicted that the activation of the amygdala and midbrain during the outcome phase would be greater than during the anticipation phase in response to a humorous reward type.

## Results

### Behavioral data

The percent of rewarded correct responses on the n-back task (combined 0-back and 2-back tasks) during scanning was 97.26% ± 8.06% (Mean ± SD) for the monetary rewards, 96.05% ± 8.71% for the humorous rewards, and 95.23% ± 12.07% for the no rewards. The percent of correct responses on the 0-back and 2-back tasks was 96.05% and 98.36% for the monetary reward, 95.07% and 97.04% for the humorous rewards, and 93.42% and 97.04% for the no rewards (see Supplementary Table S4).

The pleasure/non-pleasure ratings in response to the rewards during the scanning was 92% for the monetary rewards and 88% for the humorous rewards. To further establish that the humor stimuli effectively induced amusement or mirth, post-scan ratings using a Likert 7-point scale were conducted. In the post-scan ratings, the scores for the humorous pictures were 6.12 ± 0.71 (Mean ± SD) for comprehensibility and 5.26 ± 0.74 for funniness. The wanting scores were 5.97 ± 1.05 in the MA condition, 5.08 ± 1.17 in the HA condition, and 2.58 ± 1.22 in the NA condition in the post-scan rating. A one-way repeated-measures ANOVA of the participants’ motivation scores was significant, *F* (2, 74) = 93.89, *p* < 0.001, η_p_^2^ = 0.717. The Bonferroni post hoc tests revealed that the motivation score in the MA condition was significantly higher than in the HA condition and that the motivation score in the HA condition was significantly higher than in the NA condition. The liking scores were 5.84 ± 1.17 in the MO condition, 5.21 ± 1.21 in the HO condition, and 2.63 ± 1.40 in the NO condition. A one-way repeated-measures ANOVA of the participants’ liking scores was significant, *F* (2, 74) = 79.13, *p* < 0.001, η_p_^2^ = 0.681. The Bonferroni post hoc tests revealed that the pleasure scores in the MO and HO conditions were significantly higher than in the NO condition.

### fMRI results

#### Interaction between reward type and phase

An interaction between the reward type and phase was observed in the activation of the bilateral NAc, ACC, amygdala, and midbrain (including the periaqueductal gray, PGA). The activity in the four regions of interest (ROI) linked to the monetary and humorous rewards during the anticipation and outcome phases is shown in Tables 1 and 2.

**Table 1:** Activation levels of 4 ROIs in the brain showing the simple main effects of ‘phase’ in the monetary reward, humorous reward, and no reward control conditions.

Brain region	Anticipation phase					Outcome phase				
	MNI coordinates			Voxels	T-value	MNI coordinates			Voxels	T-value
	x	y	z			x	y	z		
**Monetary vs. no reward**	**(MA > NA)**					**(MO > NO)**				
NAc	−8	10	−2	68	7.31	−10	4	−2	22	3.58
	10	2	−2	84	7.20	6	6	0	15	3.51
ACC	−6	38	16	195	7.50	8	34	8	152	5.61
						−2	38	2	136	4.96
Amygdala	−20	−8	−10	35	4.53					
Midbrain	20	−24	−6	83	6.70					
	−8	−24	−4	184	6.51					
**Monetary vs. humorous reward**	**(MA > HA)**					**(MO > HO)**				
NAc	−12	4	0	64	6.91	10	11	−4	17	4.66
	10	6	2	75	5.89	−12	11	−2	16	4.60
ACC	4	6	−4	15	5.63	10	36	22	195	9.52
	−2	8	−4	13	4.65	−6	36	14	212	7.61
Midbrain	8	−20	−6	160	5.05					
	−8	−10	−8	12	4.30					
**Humorous vs. no reward**	**(HA > NA)**					**(HO > NO)**				
ACC	−6	34	16	39	3.60					
Amygdala	−18	−8	−14	9^†^	3.62	22	−6	−14	62	9.94
						−20	−8	−16	70	9.86
Midbrain						−4	−30	−6	239	13.04
						6	−30	−4	191	12.24
**Humorous vs. monetary reward**	**(HA > MA)**					**(HO > MO)**				
Amygdala						22	−6	−16	67	8.74
						−20	−8	−16	70	8.18
Midbrain						−6	−30	−6	215	11.66
						6	−30	−4	191	11.03

Note: The activation threshold was set to *p* < 0.05 FWE (family-wise error rate) corrected at the peak level, and clusters greater than or equal to 10 are presented. ^†^Clusters less than 10. NAc = nucleus accumbens; ACC = anterior cingulate cortex; MA = monetary reward during the anticipation phase; MO = monetary reward during the outcome phase; HA = humorous reward during the anticipation phase; HO = humorous reward during the outcome phase; NA = no reward during the anticipation phase; NO = no reward during the outcome phase.

**Table 2:** Activation levels of 4 ROIs in the brain showing the simple main effects of ‘reward type’ during the reward anticipation and outcome phases.

Brain region	Anticipation phase					Outcome phase				
	MNI coordinates			Voxels	T-value	MNI coordinates			Voxels	T-value
	x	y	z			x	Y	z		
**Monetary reward type**	**(MA > MO)**					**(MO > MA)**				
NAc	−8	12	−4	46	11.70					
	8	10	−6	71	11.19					
ACC	−4	4	−6	12	10.77					
	4	6	−6	15	10.61					
Amygdala	−26	0	−22	35	4.32					
Midbrain	−10	−18	−10	164	5.51					
	16	−10	−10	13	4.16					
**Humorous reward type**	**(HA > HO)**					**(HO > HA)**				
NAc	−8	12	−4	41	10.21					
	10	12	−4	54	9.36					
ACC	14	50	−2	74	10.57					
Amygdala						22	−6	−16	41	4.58
						−22	−8	−16	21	4.56
Midbrain						−4	−30	−4	204	11.56
						12	−28	−4	42	6.87

Note: The activation threshold was set to *p* < 0.05 FWE (family-wise error rate) corrected at the peak level, and clusters greater than or equal to 10 are presented. NAc = nucleus accumbens; ACC = anterior cingulate cortex.

#### Simple main effect during each phase

A post hoc test showed a significant simple main effect for each of the two phases. The present study was concerned with comparing the three reward types during both the anticipation (wanting) and outcome (liking) phases, particularly during the outcome phase (Table 1).

#### Anticipation phase (the ‘motivation brain’): MA vs. HA vs. NA

A post hoc test of the three conditions (monetary, humorous, and no reward) during the anticipation phase revealed activation in the left NAc, left ACC (including the subgenual area, BA 25), and the bilateral midbrain (including the PGA).

**Figure 1:**
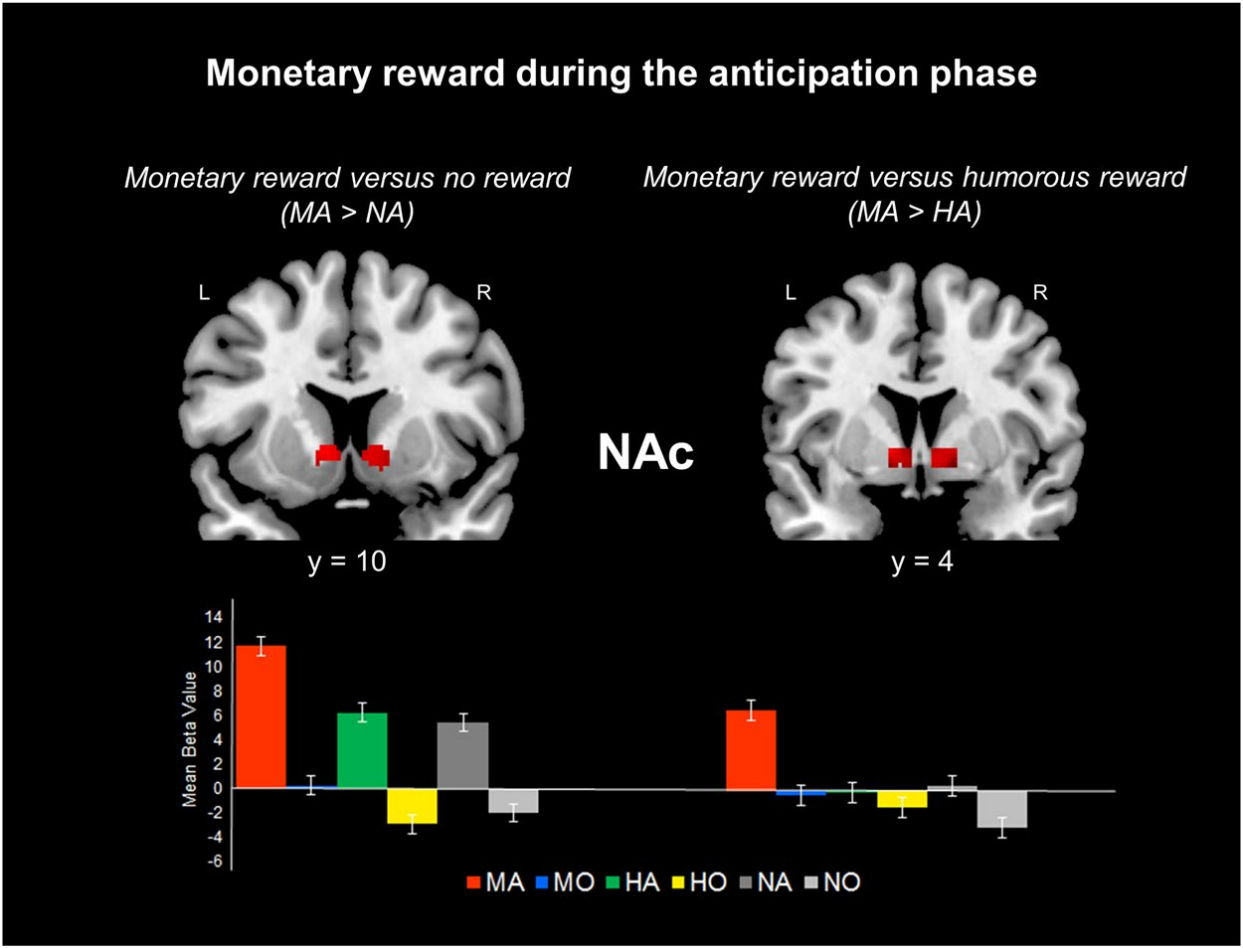
Response of the NAc to a monetary reward during the anticipation phase. (Top left) Activation foci during the MID anticipation phase and no reward control anticipation phase (MA > NA). (Top right) Activation foci during the MID anticipation phase and HID anticipation phase (MA > HA). (Bottom) Bar graphs showing the mean beta values of the peak voxels for each of the three types. Error bars indicate the SEM. MA = monetary reward during the anticipation phase; MO = monetary reward during the outcome phase; HA = humorous reward during the anticipation phase; HO = humorous reward during the outcome phase; NA = no reward during the anticipation phase; NO = no reward during the outcome phase. L = left hemisphere; R = right hemisphere.

The results during the anticipation phase are shown in Table 1 and Fig. 1. (1) The contrast between the monetary reward and no reward (baseline) conditions (MA-NA) revealed increased activation in the bilateral NAc, left ACC, left amygdala, and bilateral midbrain (including the PGA) in the monetary (MA) condition (Fig. 1). (2) The contrast between the monetary reward and humorous reward conditions (MA-HA) revealed increased activation in the bilateral NAc, bilateral subgenual ACC (BA 25), and bilateral midbrain (including the substantia nigra, PGA, and red nucleus) in the monetary (MA) condition (Fig. 1). (3) The contrast between the humorous reward and no reward conditions (HA-NA) revealed increased activation in the left amygdala and left ACC in the humorous (HA) condition. (4) The contrast between the humorous reward and monetary reward conditions (HA-MA) did not reveal any regions with increased activity in the humorous (HA) condition.

#### Outcome phase (the ‘hedonic brain’): MO vs. HO vs. NO

A post hoc test of the monetary, humorous, and no reward conditions during the outcome phase revealed activation in the bilateral NAc, right ACC, bilateral amygdala, and bilateral midbrain.

**Figure 2:**
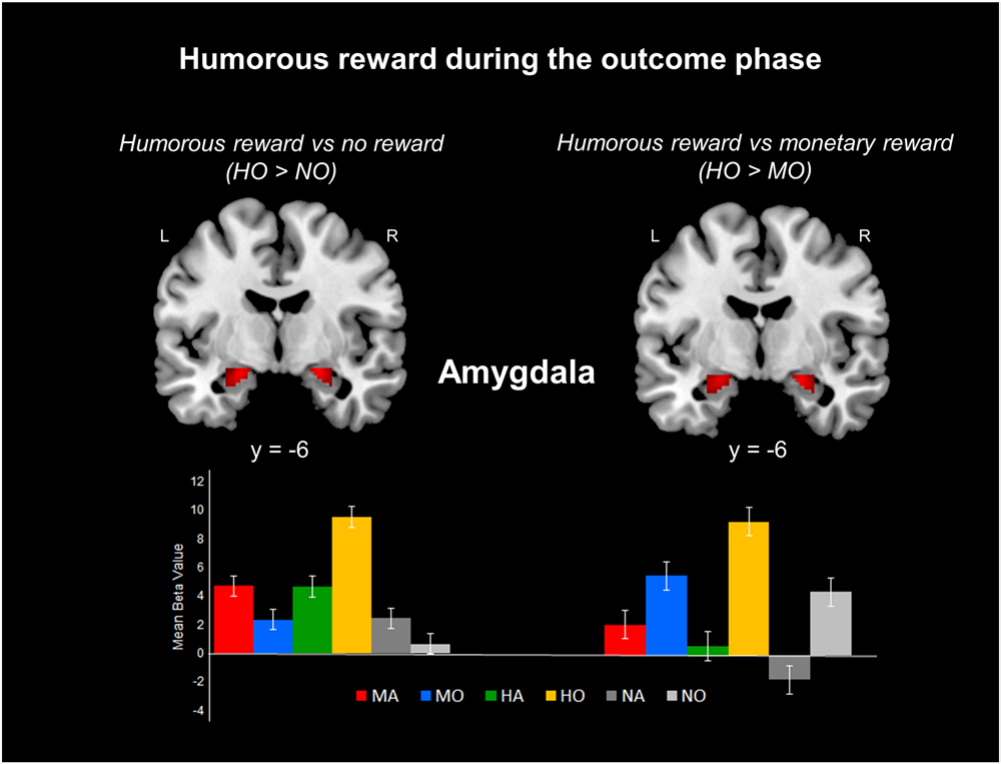
Response of the amygdala to a humorous reward during the outcome phase. (Top left) Activation foci during the HID outcome phase and no reward control outcome phase (HO > NO). (Top right) Activation foci during the HID outcome phase and MID outcome phases (HO > MO). (Bottom) Bar graphs showing the mean beta values of the peak voxels for each of the three types. Error bars indicate the SEM. L = left hemisphere; R = right hemisphere.

The results for the outcome phase are shown in Table 1 and Fig. 2. (1) The contrast between the monetary reward and no reward conditions (MO-NO) revealed increased activation in the bilateral NAc and bilateral ACC in the monetary (MO) condition. (2) The contrast between the monetary reward and humorous reward conditions (MO-HO) revealed increased activation in the bilateral NAc and bilateral ACC in the monetary (MO) condition. (3) The contrast between the humorous reward and no reward conditions (HO-NO) revealed increased activation in the bilateral amygdala and bilateral midbrain (including the PGA and red nucleus) in the humorous (HO) condition (Fig. 2). (4) The contrast between the humorous reward and monetary reward conditions (HO-MO) revealed increased activation in the bilateral amygdala and bilateral midbrain (including the dorsal and ventral PGA) in the humorous (HO) condition (Fig. 2).

#### Simple main effect of reward type

A post hoc test showed a significant simple main effect of each of the reward types (Table 2).

#### Monetary reward type: (MA vs. MO) and (MO vs. MA)

In the monetary reward conditions, the contrast between the anticipation phase and the outcome phase (MA-MO) revealed increased activation in the bilateral NAc, bilateral subgenual ACC (BA 25), left amygdala, and bilateral midbrain (including the left substantia nigra and left ventral PGA) during the anticipation (MA) phase. Conversely, in the contrast between the MO condition and MA condition, no region showed increased activation in the outcome (MO) condition (Table 2).

#### Humorous reward type: (HA vs. HO) and (HO vs. HA)

In the humorous reward conditions, the contrast between the HA and HO conditions revealed activation in the bilateral NAc and right ACC in the anticipation (HA) condition. Conversely, in the contrast between the HO condition and HA condition, increased activation in the bilateral amygdala and bilateral midbrain (including the dorsal and ventral PGA) was observed in the outcome (HO) condition (Table 2).

### Functional connectivity: psychophysiological interaction analysis (PPI)

**Figure 3:**
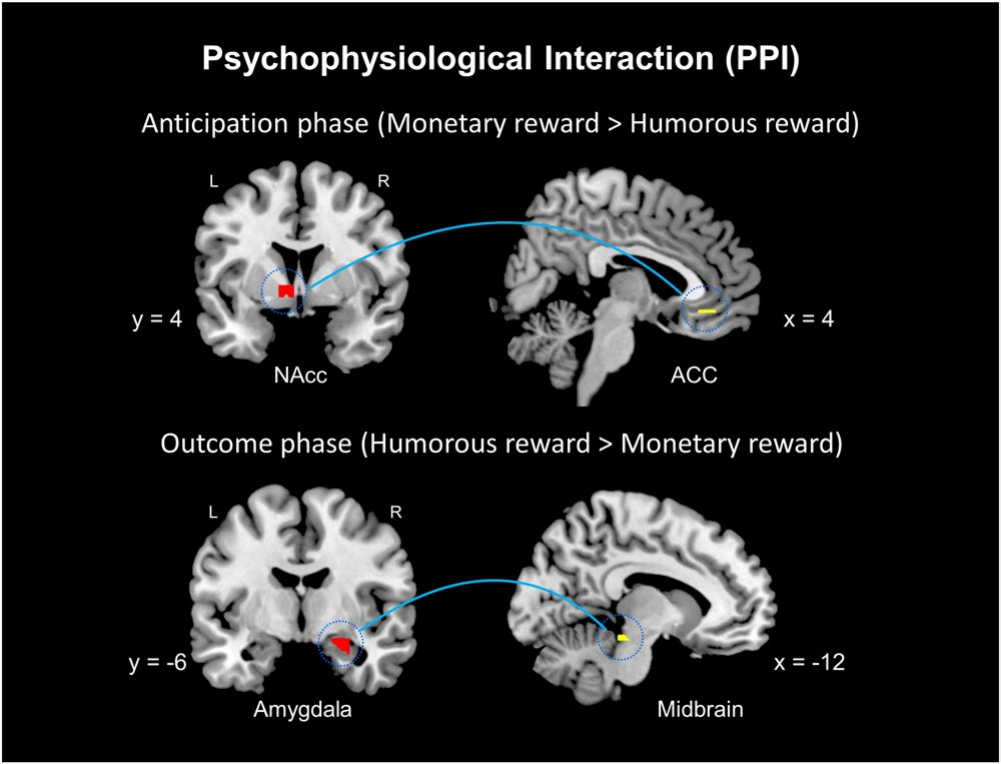
Results of the psychophysiological interaction (PPI) analysis. (Top left) The seed of the PPI analysis was placed in the left NAc (−12, 4, 0). (Top right) The effect was associated with increased NAc-ACC coupling in response to the monetary reward compared with that in response to the humorous reward (MA > HA) during the anticipation phase. (Bottom left) The seed of the PPI analysis was placed in the right amygdala (22, −6, −16). (Bottom right) The effect was associated with increased amygdala-midbrain coupling in response to the humorous reward compared with that in response to the monetary reward (HO > MO) during the outcome phase. L = left hemisphere; R = right hemisphere.

A psychophysiological interaction analysis (PPI) was conducted to determine whether an interaction exists between a psychological variable (reward type in the two phases) and the functional coupling of brain areas. The present study used the two seeds of the NAc and amygdala based on the whole mask. During the anticipation phase, the PPI analysis using the left NAc (−12, 4, 0) as a seed showed functional connectivity with the left NAc and right ACC in the contrast between the monetary (MA) condition and humorous (HA) condition. During the outcome phase, the PPI analysis using the right amygdala (22, −6, −16) as a seed showed functional connectivity with the left midbrain in the contrast between the humorous (HO) condition and monetary (MO) condition (Table 3, Fig. 3).

**Table 3:** Functional connectivity of the NAc and amygdala seeds in the psychophysiological interaction (PPI) analyses.

Brain region	Anticipation phase					Outcome phase				
	MNI coordinates			Voxels	T-value	MNI coordinates			Voxels	T-value
	x	y	z			x	y	z		
**Monetary vs. no reward**	**(MA > NA)**		**NAc seed (−8, 10, −2)**			**(MO > NO)**		**NAc seed (−10, 4, −2)**		
ACC	−8	46	−4	80	4.10	8	38	28	21	3.90
	8	46	−4	81	3.58					
Midbrain	0	−18	−6	47	3.55	18	−22	−6	21	4.37
	8	−28	−10	26	5.50					
**Monetary vs. humorous reward**	**(MA > HA)**		**NAc seed (−12, 4, 0)**			**(MO > HO)**		**NAc seed (−12, 11, −2)**		
NAc	−8	6	2	12	3.68	−8	12	−4	10	3.19
ACC	4	32	−10	16	3.28	16	46	4	13	3.99
**Humorous vs. no reward**	**(HA > NA)**		**Amygdala seed (−18, −8, −14)**			**(HO > NO)**		**Amygdala seed (22, −6, −14)**		
Midbrain	−18	−22	−12	15	2.54^†^	12	−28	−14	11	2.50^†^
**Humorous vs. monetary reward**						**(HO > MO)**		**Amygdala seed (22, −6, −16)**		
Midbrain						−12	−28	−14	11	3.45

Note: The activation threshold was set to *p* < 0.05 FWE (family-wise error rate) corrected at the peak level, and clusters greater than or equal to 10 are presented. ^†^Using the less stringent statistical threshold *p* < 0.001 uncorrected for the ROI comparison.

## Discussion

The purpose of the present study was to disentangle the MRS activity mediating monetary and humorous reward processing. The present study revealed an interaction between reward type and phase and provided evidence supporting distinct neural correlates underlying reward anticipation and outcome phases associated with monetary, humorous and no reward types. The NAc and ACC were primarily recruited during the anticipation phase in response to a monetary reward (MA-MO; MA-HA). The amygdala and midbrain were primarily recruited during the outcome phase in response to a humorous reward (HO-HA; HO-MO). To the best of our knowledge, this study is the first to find distinct patterns of reward-specific activation of the “motivation brain”, which comprises the NAc and ACC, during the anticipation phase in response to a monetary reward (MA-HA) and of the “hedonic brain”, which comprises the amygdala and midbrain, during the outcome phase in response to a humorous reward (HO-MO) (Table 1). Evidence from the functional connectivity and PPI analyses further confirmed the significant enhancements in the functional connectivity between the left NAc (seed) and right ACC regions in response to a monetary reward compared with that in response to a humorous reward during both the anticipation (MA-HA) and outcome phases (MO-HO). In addition, the PPI analysis confirmed the significantly increased functional connectivity between the right amygdala (seed) and left midbrain in response to a humorous reward compared with that in response to a monetary reward during the outcome phase (HO-MO) (Table 3). Our results identify dissociable MRS areas in response to monetary and humorous rewards during the anticipation and outcome phases.

As expected, the NAc (a key component of the MRS) was more highly activated by the anticipation of a monetary reward than by the receipt of a monetary reward (Table 2). Previous studies of the NAc in human have focused on its role in processing monetary rewards^6–8,11,12,30^ and the NAc has been consistently linked to processing reward seeking desires^6,7^. The results of the present study are consistent with these earlier findings.

Further, we found that the NAc was more robustly recruited by monetary anticipation than by humorous anticipation. Interestingly, the contrast between the monetary reward and humorous reward conditions (MA-HA) during the anticipation phase revealed increased activation in the NAc and subgenual ACC (sgACC, BA 25) in the MA condition (Fig. 3). This observed sgACC activation is inconsistent with previous studies finding activation during the anticipation of monetary rewards in the dACC (BA 32)^8,16^. Activation in the dACC is known to play a role in value-guided choices^16,31^. The participants in our study may have been motivated by their desire to gain monetary rewards in a situation in which, unlike some of the earlier studies, no chance of a penalty or monetary loss was presented and participants were thus not required to make a choice.

Finally, the contrast between the humorous reward condition and monetary reward condition (HA-MA) during the anticipation phase did not show significant activation in any area in the HA condition. Altogether, monetary and humorous rewards may differentially recruit distinct MRS components that project primarily to the NAc and sgACC during the anticipation phase. To the best of our knowledge, this study is the first to demonstrate the dissociative activation of the NAc and sgACC in humans anticipating a monetary but not a humorous reward.

In contrast with the anticipation phase, reward consumption evoked different patterns of activation in response to humorous and monetary rewards. The contrast between the humorous reward conditions and monetary reward conditions during the reward outcome phase revealed a significant activation of the amygdala and midbrain (Fig. 2) in the humorous reward condition.

This is consistent with previous findings that the amygdala and midbrain contribute to hedonic feelings of humor appreciation for both visual humor (sight gags) and language-based humor^23^. The amygdala is considered a central component of the MRS and has repeatedly been implicated in feelings of amusement^19^. The amygdala plays a key role in the regulation of feelings of amusement during humor appreciation and the receipt of social rewards via dopaminergic projections from the midbrain. The observed amygdala activation is consistent with previous studies examining the hedonic consumption of humor^13,18–24^, social rewards^12^, erotic pictures^32^, and monetary rewards^33^. The present finding of pronounced amygdala and midbrain activation during the receipt of humorous rewards compared to that during the receipt of monetary rewards could be attributed to the appreciation and enjoyment of humorous stimuli.

Perhaps more surprisingly, compared to the consumption of monetary rewards, the NAc and ACC were not found to be activated during the consumption of humorous rewards. The NAc has been shown to be a key component of the MRS in a series of humor studies^19,22^. Previous humor studies, comparing funny and nonfunny cartoons, indicated that humor engages a network of subcortical structures, including the midbrain (ventral tegmental area, VTA), NAc and amygdala, and that the NAc activation reflects hedonic amusement^19^. Our previous studies used verbal jokes as stimuli and also showed activation in the amygdala, midbrain, NAc and ACC^13,18,20^. In contrast with these findings, the present study did not find increased NAc activity for humor rewards versus monetary rewards in the outcome phase. One interpretation of these results would be that sensitivity to the rewarding effects of monetary gains in the NAc may be greater than for the rewarding effects of humor appreciation. Humor appreciation requires an elaboration stage to associate humorous attributes to the characters in the joke, thereby generating feelings of amusement^20,21^. The present study utilized humorous pictures (single-frame and captionless cartoon images) instead of verbal jokes, and the use of humorous pictures might require less elaboration than the characters in a verbal joke.

Conversely, the contrast between the monetary reward outcome and humorous reward outcome revealed significant activation in the NAc and dorsal ACC (dACC, BA 32). The ventral striatum, specifically the NAc, is activated during reward anticipation but not significantly more than during consumption^8^. However, our results showed that the NAc and ACC (sgACC and dACC) were involved in both the pursuit of monetary rewards and the hedonic consumption of receiving monetary rewards. The finding of pronounced NAc and ACC activation during consumption of a monetary reward highlights the recruitment of the NAc and ACC during the anticipation and receipt of a monetary but not of a humorous reward.

The ACC has been implicated in reward motivation and reward learning^34^. The contrast between the monetary reward condition and humorous reward condition (MA-HA) during the anticipation phase revealed increased activation in the bilateral subgenual ACC (sgACC, BA 25) in the monetary reward condition, while the contrast between the monetary reward condition and humorous reward condition (MO-HO) during the outcome phase revealed increased activation in the bilateral dorsal ACC (dACC, BA 32) in the monetary reward condition. The sgACC has been implicated in the automatic modulation of affect^34^, while the dACC integrates information regarding executive functions and cognitive control^8^. Thus, one possible interpretation of our findings would be that sgACC activity is associated with prediction of monetary reward and reflects motivational and affective aspects of reward processing in sensitivity to monetary value coding and retaining the reward value. The MID task was developed to induce motivational and affective reward processing by using rapid presentation of reward cues and invoking contingency^35^. The present study may have invoked more cognitive and deliberative monetary reward valuations, as participants perceived that their n-back performance would determine their outcomes (i.e., contingency). On the other hand, increased cognitive processing may be more likely to recruit dorsal components of reward circuitry. The dACC has been implicated in cognitive processes that play a core role in integrating information to select or modify appropriate motor responses and to link reward-related information with alternative actions^36^. The present study showed New Taiwan dollar signs, indicating to the participants that they had earned real cash rewards. The dACC activity found may have been associated with outcome evaluation during this presentation of monetary signs.

Altogether, monetary rewards generate pleasure or enjoyment via NAc-ACC connectivity, while the consumption of humorous rewards induces feelings of amusement generated by reward circuits involving amygdala-midbrain connectivity (Fig. 3). Thus, the neural circuits underlying the hedonic consumption of monetary and humorous rewards are largely distinct.

The present study also helped disentangle the neural networks mediating the anticipatory and consummatory aspects of reward processing. Previous animal and human studies have shown that dopamine is more robustly released in the NAc during reward anticipation than during reward consumption^1,6–8^. The finding in the present study of activation in the NAc and ACC during the anticipation phase is greater than during the consumption phase in response to both monetary and humorous rewards (MA-MO; HA-HO) is thus consistent with these earlier studies.

The percentage of accurate responses for the 0-back tasks was higher than for the 2-back tasks, but by applying the two criteria (75% and 50%), the pattern was reversed (Supplementary File, Section 3.4 and Table S4). Reaction times on the successful 0-back tasks in each of the three conditions were significantly shorter than reaction times on successful 2-back tasks. However, reaction times for unsuccessful 0-back tasks in the no reward condition were significantly longer than those for unsuccessful 2-back tasks in all three conditions (monetary, humorous and no reward). In the post-scan debriefing, some participants reported feeling that the 2-back tasks were more challenging and generated higher motivation to earn the rewards, especially in the monetary reward condition. In contrast, the 0-back tasks were much easier, and participants might thus have been more easily distracted due to being overly relaxed. These possibilities should be considered in future studies.

In conclusion, our findings demonstrate that the brain response to monetary and humorous rewards can be dissociated in both the ‘motivation brain’ and the ‘hedonic brain’. Evidence from functional connectivity through PPI analyses further confirmed significant enhancements in functional connectivity between the left NAc and right ACC regions in response to a monetary reward compared with responses to a humorous reward during both the anticipation and outcome phases, while no region showed significant activation in response to the humorous reward during the anticipation phase. Importantly, our results revealed significantly increased functional connectivity between the right amygdala and left midbrain in response to a humorous reward compared with that in response to a monetary reward (HO-MO) during the outcome phase, while monetary rewards elicited greater activation in the left NAc and right ACC (MO-HO) during the outcome phase. In addition, the current study showed increased activation in the NAc and ACC during the anticipation phase compared with the consumption phase.

Future studies could further investigate the common and distinct brain networks processing the hedonic experiences of primary (e.g., erotic rewards) and secondary rewards (monetary and humor rewards). Future studies may also examine the neural basis of anhedonia in schizophrenia during in-the-moment hedonic experience (liking) phase for humor and monetary rewards.

## Methods

### Participants

Initial participants included forty-three right-handed healthy volunteers (21 men and 22 women) with no history of neurological or psychiatric problems. All participants took part in all three conditions. The responses for five participants (2 men and 3 women) were excluded from analysis for failing to meet the inclusion criteria of the n-back task (see below). Thus, the results for 38 participants (19 men and 19 women; mean age = 23.34 ± 2.14; range = 20–30 years) were included in the study. All experimental protocols performed in this study were approved by the Research Ethics Committee of National Tsing Hua University. All experiments were performed in accordance with the relevant guidelines and regulations. Written informed consent was obtained from all participants prior to participation.

### Tasks and stimuli

The experiment consisted of the following two incentive delay tasks: the monetary incentive delay (*MID*) task and the revised humor incentive delay (*HID*) task. The two tasks were designed to capture both the ‘anticipation’ phase of incentive motivation and the ‘outcome’ phase of hedonic pleasure upon receiving a reward, but the tasks differed in terms of the stimulus type (monetary versus humorous) used. In the no reward task, scrambled pictures were used as a no-feedback control. The present study used a modified MID task with an n-back task instead of a simple judgment task.

To find and select appropriate humor stimuli, two pilot studies were conducted. The sixteen best, single-frame, captionless cartoon images were selected from 600 cartoons using the behavioral results of the pilot studies. The procedure used to select the humor stimuli and the results are described in greater detail in the supplementary information (see Supplementary Table S1). The present study utilized the 16 most salient humorous images based on the behavioral results from the pilot studies. Forty-eight trials were performed, including sixteen trials in each of three conditions: monetary reward, humorous reward, and no reward conditions.

### Experimental paradigm

**Figure 4:**
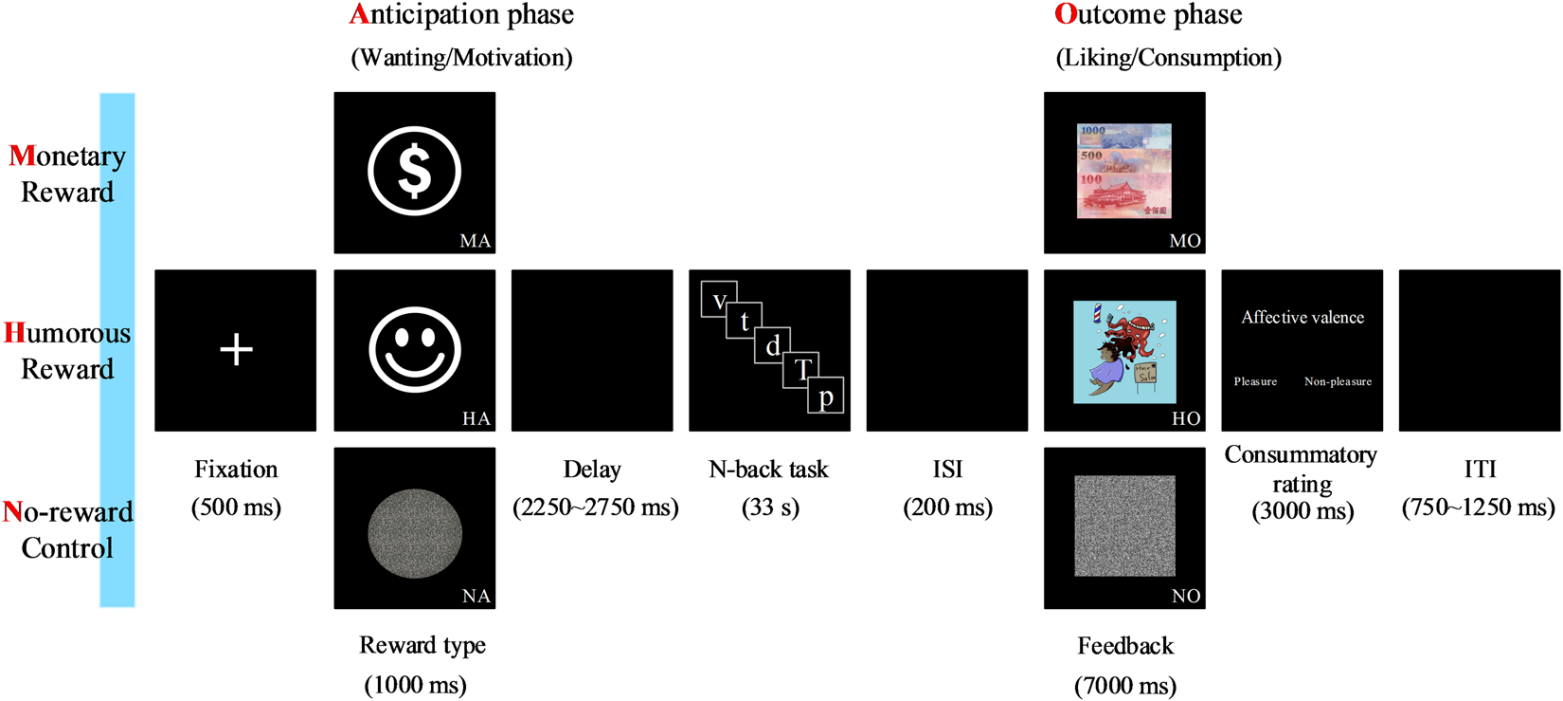
Experimental paradigm for the monetary reward, humorous reward, and no reward control conditions during the anticipation and outcome phases. Trials of monetary incentive delay (MID) and humorous incentive delay (HID) tasks in the monetary, humorous, and no reward control conditions. Reward processing involves both the anticipation and outcome phases. Each trial consists of a reward type, an anticipation delay period, an n-back task, a feedback display (MID, HID, and no reward feedback), and a consummatory rating.

The present study employed a 3 × 2 repeated-measures factorial design with reward type (monetary vs. humorous vs. no reward) and reward phase (anticipation vs. outcome) as factors, yielding a total of six conditions as follows: MA, MO, HA, HO, NA, and NO (Fig. 4). Among them, the no reward conditions (NA and NO) were not only experimental conditions, but also the baseline in the current paradigm. The following six primary conditions (3 types × 2 phases) were defined: “monetary reward during the anticipation phase” (MA), “monetary reward during the outcome phase” (MO), “humorous reward during the anticipation phase” (HA), “humorous reward during the outcome phase” (HO), “no reward control during the anticipation phase” (NA), and “no reward control during the outcome phase” (NO).

At the beginning of each trial, a fixation was presented for 500 ms. The participants were presented with one of the three reward types (monetary, humorous, and no reward control) for 1000 ms at the center of the computer screen. The reward was followed by a delay lasting between 2250 ms to 2750 ms, which was designed to retain the wanting (motivation) during the anticipation phase. Then, an n-back task (0-back and 2-back) appeared, lasting for 33 s. The participants were encouraged to respond correctly to all cues, each of which was followed by an interstimulus interval (ISI) of 200 ms. The reward outcome phase lasted 7000 ms. Participants were first notified of whether they had gained the reward according to their performance on the n-back task during that trial and were then shown the reward outcome. Thus, successful trials were associated with monetary gains and humorous amusement, while unsuccessful trials led to a scrambled picture as a non-reward. In the no reward condition, participants saw scrambled pictures regardless of whether their performance was successful or unsuccessful. The outcome phase of the MID and HID tasks lasted for 7000 ms to facilitate the emotion-eliciting and amusement-eliciting effect of the affective images, respectively. The participants provided a subjective affective valence judgment within 3 s by pressing one of two buttons on a response box (Lumina, LS-PAIR) under their right index or middle fingers, and an intertrial interval (ITI) ranging between 750 ms to 1250 ms followed.

The n-back task paradigm includes the 0-back and 2-back tasks. There were three different types of reward conditions: monetary rewards, humorous rewards, and no rewards, with each reward condition containing 16 trials for a total of 48 trials. In each reward condition, both the 0-back and 2-back tasks appeared eight times (i.e., eight trials for each n-back in each condition). For the 0-back trials, participants needed to respond to the target that was identical to the pre-specified letter (“X”). For the 2-back trials, participants needed to respond to the target that was identical to the letter that appeared two letters before the letter currently shown. All the letters in the n-back task were drawn from the following seven consonants: p, g, b, d, t, v, and c. The n-back task, procedure, and the results are described in greater detail in the supplementary information (see Supplementary Fig. S3).

Each trial of the n-back task was composed of 12 letters, and 4 out of 12 letters (33%) in a given trial were targets^37^, while the other 8 letters were non-targets. The current study was designed to examine performance in the outcome phase of reward processing. We set two criteria for getting a reward: (1) the percentage of accurate responses for targets and non-targets had to be greater than or equal to 75% (i.e., correct responses to at least 9 out of 12 letters), and (2) the percentage of accurate responses for targets had to be greater than or equal to 50% (i.e., correct responses to at least 2 out of 4 target letters). As noted, in the no reward condition, participants saw scrambled pictures in both the 0-back and 2-back tasks regardless of whether their performance was successful or unsuccessful. Data from both the monetary and humorous reward conditions were included in the analysis only if at least 12 out of 16 trials in each reward condition met the above criteria (see Supplementary Fig. S4 and Table S4).

During the MID task, the participants received monetary pictures as feedback for successful trials. If the participants succeeded on at least 12 out of 16 trials, the 200 New Taiwan dollars (approximately 6.50 US dollars) they won became a part of the monetary compensation they received after the experiment. During the HID task, the participants received humorous pictures as feedback during the outcome/consumption phase.

The experimental tasks were presented using E-Prime 2.0 software (Psychology Software Tools, Inc., Pittsburgh, PA) to present the stimuli. Four functional runs were performed. Each run consisted of 12 trials, including 4 trials of each of the three following conditions: monetary, humorous, and no reward. The four runs in each sequence were presented in a counterbalanced order (Latin square design) across participants.

### Image acquisition

The imaging was performed using a 3 Tesla Siemens Magnetom Prisma scanner (Erlangen, Germany) using standard gradients and a standard 20-channel head coil at the Imaging Center for Integrated Body, Mind and Culture Research in Taiwan. The visual stimuli were presented via MRI-compatible goggles (Resonance Technology, Inc.). Thirty-six interleaved slices parallel to the anterior commissure-posterior commissure line were acquired per volume. The functional scans were acquired using a T2*-sensitive gradient echo sequence with the following parameters: repetition time (TR) = 2000 ms, echo time (TE) = 30 ms, flip angle (FA) = 90°, matrix size = 64 × 64, field of view (FOV) = 220 mm × 220 mm, slice thickness = 3.40 mm, and voxel size = 3.43 × 3.43 × 3.40 mm^3^ (no gap) for the measurement of the BOLD effect. The initial three dummy scans were discarded to allow for the T1 equilibration effect. This plane of acquisition and voxel size provided adequate resolution of the ROI, including the NAc, ACC, amygdala, and midbrain. The structural scans were acquired using a T1-weighted sequence with the following parameters: TR = 1900 ms, TE = 2.28 ms, FA = 9°, matrix size = 256 × 256; FOV = 256 mm × 256 mm, number of slices = 192, slice thickness = 1 mm, and voxel size = 1 × 1 × 1 mm^3^. The structural scans facilitated the localization and co-registration of the functional data. During each run, a series of 292 EPI-scans, lasting approximately 9 min and 39 s with a 2-min break between runs, was acquired. The total duration of the experiment was approximately 44 min and 58 s per participant.

### Image analysis

The analyses focused on the changes in the BOLD contrast that occurred during the anticipation and outcome phases during each trial. The data preprocessing and analysis were performed using SPM12 (Wellcome Department of Cognitive Neurology, University College London). During the preprocessing, the functional images were slice-time corrected to the onset of the middle slice and spatially realigned to correct for head motion. Then, the high-resolution T1 images were co-registered with the functional images. The functional images were spatially normalized to the Montreal Neurological Institute (MNI, McGill University, Montreal, Quebec, Canada) space and subsequently smoothed with a Gaussian kernel of 8 mm full-width at half-maximum (FWHM).

The data analysis was performed using GLM in the first-level analysis. The presentation of reward type and phase was modeled by convolving the stimulus onset using the canonical hemodynamic response function (HRF), and the six conditions (MA, MO, HA, HO, NA, and NO) and six head movement parameters as covariates of no interest were modeled separately for each task and each run. For each participant, contrast images coding the condition onset were constructed. Then, these contrast images were entered into a second-level random effects analysis. A flexible factorial analysis was performed to determine the differences in brain activation among the three reward types (monetary, humorous and no reward) and two phases (anticipation and outcome). Although the anticipation phase was explicitly modeled in our analysis, we also focused on the ‘outcome phase’ because our interest was the differences in the coding of the hedonic enjoyment of consumption between monetary and humorous reward feedback. To compare the monetary and humorous rewards during the outcome phase, the inclusion criteria were set such that each participant’s responses were included in the study only if they succeeded on at least 75% of the n-back responses during each trial (nine successful judgments out of 12 consonant letters), at least 50% of the responses for target letters (2 successful judgments out of 4 targets), with the number of successfully rewarded trials greater than 4 for both the 0-back and 2-back tasks trials (out of 8 trials for both in each condition), and succeeded in at least 75% of the trials in each condition (receiving 12 successful feedbacks in 16 trials in each condition). Therefore, the analysis included at least 12 trials with successful performance on the n-back tasks for each reward type and each participant.

The mask of reward motivation and hedonic emotion was associated with brain regions in the predefined ROIs. Specifically, the analyses focused on four masks in the ventral striatum (NAc), anterior cingulate cortex (ACC), amygdala, and midbrain. An ROI statistical analysis was performed to test a specific a priori hypothesis. Anatomical ROIs of midbrain, amygdala, and ACC were defined by WFU PickAtlas Tool (http://www.fmri.wfubmc.edu), which generates ROI masks. Since the NAc is not contained within the WFU PickAtlas Toolbox, the NAc mask was defined in SPM12 by WFU PickAtlas Tool. The boundaries of the NAc ROI were defined according to previous studies^38,39^. The present study used a mask that ranged from (x: ±3.75 to 15.1; y: 0 to 11; z: 2.2 to −10.2) with a 6 mm radius sphere centered around a specified coordinate (MNI coordinate: ±9.425, 5.5, −4.2).

The NAc has previously been implicated in the wanting of a reward^1,6–8^, and the amygdala has been implicated in the feeling of amusement in humor studies^13,18–24^. We analyzed the functional coupling between the left NAc and right amygdala as seeds using a PPI analysis^40^.

In all analyses, the threshold was set to *p* < 0.05, and the peak level was corrected for multiple comparisons using the family-wise error rate (FWE) with at least 10 contiguous voxels after small volume correction (SVC) on anatomical ROIs.

## Electronic supplementary material

Supplementary Information
