# Supplementary material for: Dissociation between the processing of humorous and monetary rewards in the ‘motivation’ and ‘hedonic’ brains
Source: Sci Rep. 2018 Oct 18;8:15425. doi: 10.1038/s41598-018-33623-4 (PMC6194129; doi:10.1038/s41598-018-33623-4)
Supplement: Supplementary file 1 — Supplementary Information [file 41598_2018_33623_MOESM1_ESM.pdf]

Dissociation between the processing of humorous and monetary rewards in the 'motivation' and 'hedonic' brains

Yu-Chen Chan[1*, 2] Wei-Chin Hsu[3] Tai-Li Chou[4]

[1]Department of Educational Psychology and Counseling, National Tsing Hua University, Hsinchu, Taiwan

[2]Research Center for Education and Mind Sciences, NTHU

[3] Graduate Institute of Applied Science and Technology, National Taiwan University of Science and Technology, Taipei, Taiwan

[4] Department of Psychology, National Taiwan University, Taipei, Taiwan

Supplementary information

1. Behavioral study I: Select stimuli

To ensure that the humorous pictures were valid stimuli, two behavioral studies were conducted prior to the fMRI experiment. In the first study, 1,886 humorous images and 139 neutral images were selected from the Internet. The humorous images included single-frame, caption-less cartoon images designed to elicit humor-related cognitive and affective processes. The neutral images included every-day, simple

objects, such as flowers, chairs, dogs, and houses. The humorous and neutral images used as stimuli were chosen by a group of five judges, all with experience in humor research. Each image was reviewed. Images with significant overlap or duplication of content were removed. An initial set of 600 humorous and 40 neutral images was selected. All the images were 16 × 16 cm, or with at least one side that was 16 cm long. The resolution of these images was at least 300 dpi, and they were presented on the screen at a proportion of 4 × 3. Examples of a humorous stimulus and a neutral stimulus are shown in Figure S1.

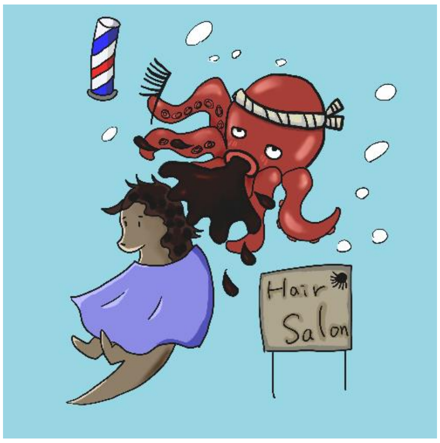

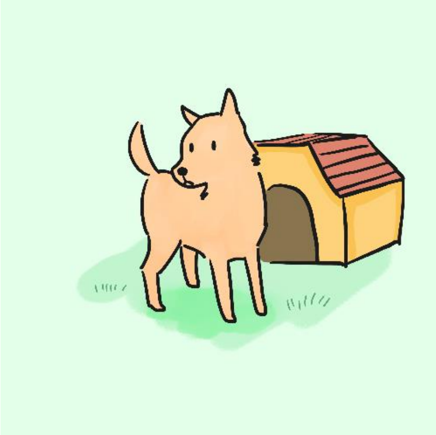

Figure S1 (Left) humorous stimulus. (Right) neutral stimulus. The images were created by Hsin-Yu Kuo, based on ideas that originated with the corresponding author's team. The authors obtained permission to use the images.

One hundred and eight participants (44 men, ages ranging from 18 to 28 years, mean age of 21.82 ± 1.92 years) rated each joke based on its degree of comprehensibility and funniness on a 7-point scale using E-Prime 2.0 software. Each

participant was presented with 100 humorous and 40 neutral images. Four runs of images were performed, and the runs were presented in a counterbalanced order across participants with at least a 1 min break between runs. The total duration of the experiment was approximately 30 min. The means and standard deviations of the reaction times (RTs) of comprehensibility and the rated levels of comprehensibility and funniness in the two different conditions are shown in Table S1. A one-way repeated-measures ANOVA of the participants' funniness ratings was significant, F (1, 107) = 542.09, $p < .001$, $\eta_p^2 = .835$.

Table S1 Means and standard deviations of the reaction times of comprehensibility and ratings of comprehensibility and funniness (N = 108)

	Reaction time (ms)		Comprehensibility		Funniness	
	M	*SD*	*M*	*SD*	*M*	*SD*
Humorous	7277.78	2693.06	6.35	.44	4.03	1.16
Neutral	3424.98	1993.02	6.53	.51	1.47	.74

Note: In total, 600 humorous images and 40 neutral images were scored

Based on the results of this behavioral study, in the present study, the 16 most salient humorous images were selected. Sixteen humorous images with RTs less than

7 s and scores above 6 for comprehensibility and above 5 for funniness were selected. The mean and standard deviation of the RT for comprehensibility was 4800.01 ± 1009.54 ms, of the comprehensibility ratings was 6.83 ± 0.11, and of the funniness ratings was 5.72 ± 0.21 (Table S2).

Table S2 Means and standard deviations of the reaction time of comprehensibility and ratings of comprehensibility and funniness ($N = 108$)

	Reaction time (ms)		Comprehensibility		Funniness	
	M	*SD*	*M*	*SD*	*M*	*SD*
Humorous	4800.01	1009.54	6.83	.11	5.72	.21

Note: The 16 most salient humorous stimuli were selected

2. Behavioral study II

A second study involving a separate group of 64 participants (31 men, ages ranging from 18 to 27 years, mean age of 21.45 ± 1.93 years) was conducted. The 16 most salient humorous images were used. The purpose of this study was to compare the impact of monetary rewards, humorous rewards, and no reward on cognitive and affective processing and to better distinguish the effects of reward anticipation (wanting) from those of reward outcome (liking). The second study utilized the 16

most salient humorous images from the initial behavioral results. Forty-eight trials were performed, including sixteen trials of each of the following three different conditions: monetary reward, humorous reward, and no reward.

The procedure is shown in Figure S2. The experiment consisted of the following three tasks: the monetary incentive delay task (MID), the revised humorous incentive delay task (HID), and the no reward task. Each task consisted of 16 trials. All participants viewed and rated each trial for wanting and liking on a 7-point scale using E-Prime 2.0 software. The means and standard deviations of the rated levels of wanting and liking in all three conditions (16 trials each) are shown in Table S3.

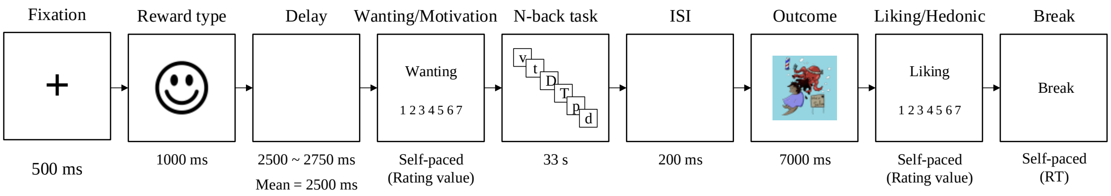

Figure S2 The trial procedure used in the behavioral study

A one-way repeated measures ANOVA of the participants' wanting scores was significant, F (2, 126) = 172.07, $p < .001$, $\eta_p^2 = .732$, and the Bonferroni *post hoc* tests revealed that the monetary reward condition received significantly higher wanting (motivation) scores than the humorous and no reward conditions. A one-way repeated measures ANOVA of the participants' liking scores was significant, F (2, 126) =

169.53, $p < .001$, $\eta_p^2 = .729$, and the Bonferroni *post hoc* tests revealed that the monetary reward condition received significantly higher liking (hedonic impact) scores than the rewards in the other conditions.

Table S3 Means and standard deviations of the wanting and liking scores ($N = 64$)

	Wanting		Liking	
	M	*SD*	*M*	*SD*
Monetary reward	6.12	.94	5.99	.96
Humorous reward	5.31	1.43	4.95	1.11
No reward control	2.37	1.36	2.49	1.42

3. fMRI study: N-back task

3.1 N-back task

A general description of the n-back tasks is provided in the Experimental paradigm sub-section of the Methods section in the manuscript. Figure S3 provides a visual illustration to support that description. For the 0-back trials, participants needed to respond to the target that was identical to a pre-specified letter (“X”). For the 2-back trials, participants needed to respond to the target that was identical to the letter that appeared two letters previously (Figure S3).

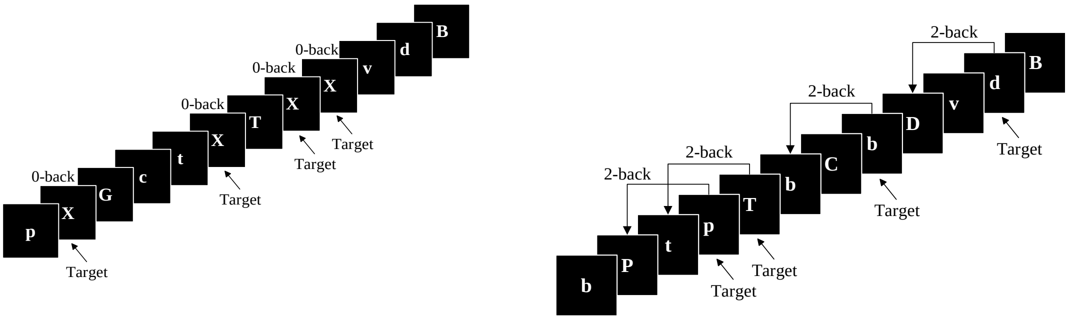

Figure S3 N-back task. (Left): 0-back task (control baseline). (Right): 2-back task (working memory task).

3.2 N-back procedure:

The present study used only two tasks—a working memory task (2-back) and control task (0-back). Participants observed stimuli on a visual display and provided n-back task judgments with a response box (Lumina, LS-PAIR). For each trial, an n-bask task instruction (0-back or 2-back cues) appeared, lasting for 2000 ms. The n-back task was followed by a delay lasting for 1000 ms. Stimuli were presented centrally with 500 ms duration, followed by a 2000 ms interstimulus interval (ISI). Participants needed to respond by pressing one of two buttons on a response box with their dominant right index for targets (4 times in a trial) or middle fingers for non-targets (8 times in a trial) after the letter disappeared (see Supplementary Figure S4). The stimulus onset asynchrony (SOA) of per letter was 2500 ms (500 ms + 2000 ms). Each n-back trial lasted for 30 s (2500 ms × 12 letters). With the 2000-ms instruction

cue and 1000-ms retention delay, the total duration of a given trial is 33 s.

During the fMRI acquisition, participants were required to perform four separate runs. Each run consisted of 12 n-back trials. Each run was composed of two 0-back trials and two 2-back trials of each condition (monetary, humorous, and no reward conditions).

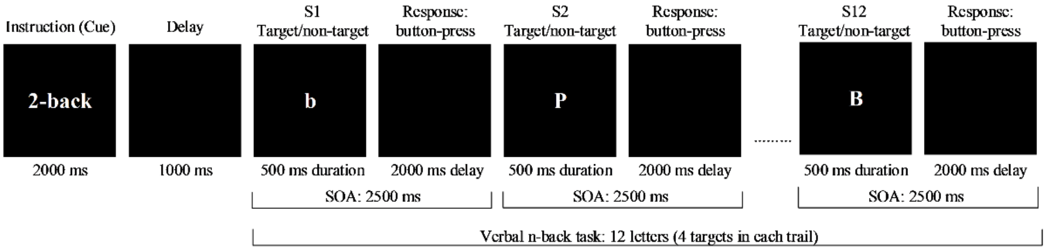

Figure S4 Experimental paradigm of N-back task. Each trial consists of an instruction cue, a retention delay, and an n-back task.

3.3 Analysis of n-back task

(A) Percentage of accurate responses

The highest possible score for a given trial was 12. A correct response was given 1 point, whereas incorrect responses did not receive any points. There were 48 trials in the experiment, so the highest possible score was 576 (12 × 48 trials). The total scores participants received were converted into percentages of accurate responses.

(B) Two Criteria: successful or unsuccessful trials.

As the current study focused on the performance of reward outcomes, we set the following two criteria: (1) the total score of the responses in each trial should be greater than or equal to 9, and (2) the total score of the responses for targets in each trial should be greater than or equal to 2. If both of the criteria were met, participants saw the reward pictures, and the trial was counted as a successfully rewarded trial. We then calculated the total number of successfully rewarded trials. The total number of trials that failed to receive rewards was calculated by subtracting the total number of successfully rewarded trials from the 48 total trials in the experiment for each participant (Table S4).

3.4 Results of n-back task

(A) Percentage of accurate of responses and two criteria

The percentage of accurate responses for the 0-back tasks was higher than for the 2-back tasks (see the upper part of Table S4). By applying the two criteria (75% and 50%), the pattern was reversed (see the bottom part of Table S4). This is because only 6 out of 38 participants reached at least 70% of accurate responses in the 0-back task in some given trials. However, they did not meet our two criteria, and yielded a smaller number of successfully rewarded trials in the 0-back task.

Only participants with more than 12 successfully rewarded trials (out of 16

trials in the experiment), and with the number of successfully rewarded trials larger than 4 in both the 0-back and 2-back tasks trials (out of 8 trials for both in each condition), were included in the present fMRI study.

(B) Reaction times on responses to the n-back task

This study consisted of three conditions, a monetary condition, a humorous condition, and a no reward condition. Each condition included eight 0-back trials and eight 2-back trials, for a total of 16 trials per condition. Each trial consisted of the presentation of 12 letters as stimuli. Each letter was presented in the center of the screen for 500 ms. After the 500 ms, the letter disappeared, and participants had 2000 ms to respond with their judgment. Participants responded by pressing one of two buttons on a response box with their (dominant) right hand to indicate whether the letter was a target or a non-target letter. The reaction time of judgment responses was recorded. Within each condition, participants thus made 96 judgments for each of the two n-back types (12 letters per trial, eight trials for each task type per condition). When participants failed to respond, a reaction time of 2000 ms was recorded. The reaction times were thus calculated using 96 judgment responses (whether successful or unsuccessful) for both 0-back and 2-back tasks within each of the three conditions. A summary of the data on reaction times is provided in Table S4.

A one-way repeated measures ANOVA of reaction times on successfully rewarded trials was significant, F (5, 185) = 50.97, $p < .001$, $\eta_p^2 = .579$, and the Bonferroni *post hoc* tests revealed that the reaction time on the successful 0-back responses in each of the three conditions (monetary, humorous, and no reward) were significantly shorter than the reaction times on the successful 2-back responses. For the successful 2-back responses, the reaction times in the monetary reward condition were significantly shorter than for the no reward condition. There was no significant difference in the reaction times in the monetary and humorous conditions on successfully rewarded 0-back tasks, nor for 2-back tasks.

A one-way repeated measures ANOVA of the participants' reaction time on unsuccessfully rewarded trials was significant, F (5, 60) = 10.01, $p < .001$, $\eta_p^2 = .455$, and the Bonferroni *post hoc* tests revealed that reaction times on the unsuccessful 2-back responses in the monetary condition were significantly shorter than the unsuccessful 0-back responses in the other three conditions (monetary, humorous and no reward) and that the unsuccessful 0-back responses in the no reward condition were significantly longer than unsuccessful 2-back responses in the other three conditions (monetary, humorous and no reward). There was no significant difference between the 0-back tasks on unsuccessfully rewarded trials across the three conditions.

A one-way repeated measures ANOVA of reaction times on all rewarded trials was significant, F (11, 132) = 70.08, $p < .001$, $\eta_p^2 = .854$, and the Bonferroni *post hoc* tests revealed that reaction times on all successful n-back responses were significantly shorter than the reaction times on all unsuccessful n-back responses. The reaction times on unsuccessful 0-back tasks in the no reward condition were significantly longer than all the other n-back responses, with the exception of 0-back tasks in the unsuccessful monetary and humorous reward conditions.

In summary, the percentage of accurate responses for the 0-back tasks was higher than for the 2-back tasks, but by applying the two criteria (75% and 50%), the pattern was reversed. Reaction times on the successful 0-back tasks in each of the three conditions were significantly shorter than reaction times on successful 2-back tasks. However, reaction times for unsuccessful 0-back tasks in the no reward condition were significantly longer than those for unsuccessful 2-back tasks in all three conditions (monetary, humorous and no reward).

According to the behavioral and fMRI results, the percentage of accurate responses was higher for the 2-back than for the 0-back tasks after applying the two criteria. In the post-scan debriefing, some participants reported feeling that the 2-back tasks were more challenging and generated higher motivation to earn the rewards in each condition, especially in the monetary reward condition. In contrast, the 0-back

tasks were much easier, and participants might thus have been more easily distracted due to being overly relaxed. They may have, for example, accidentally pressed the wrong button or missed responses. These possibilities should be considered in future studies.

Table S4 Percentage of accurate of responses and meeting the two criteria on the n-back task (N = 38)

	Monetary reward		Humorous reward		No reward control	
	0-back	2-back	0-back	2-back	0-back	2-back
Percentage of accurate responses						
Mean (accuracy)	95.91%	94.82%	95.86%	94.40%	95.10%	93.48%
Standard deviation (SD)	7.43%	4.73%	7.30%	4.06%	8.19%	5.87%
Reward type (Mean ± SD)	95.46% ± 5.34%		95.19% ± 4.97%		94.29% ± 6.23%	
Two Criteria (75% and 50%)						
No. of successfully rewarded trials	292	299	289	295	284	295
Percentage of successfully rewarded trials	96.05%	98.36%	95.07%	97.04%	93.42%	97.04%
Mean percentage of successfully rewarded trials	97.20%±8.06%		96.05%±8.71%		95.23%±12.07%	
Mean (RT)	401.58	500.04	392.08	515.34	386.73	530.55
Standard deviation (SD)	16.53	19.23	16.97	19.68	17.24	20.31
No. of trials not receiving rewards	12	5	15	9	20	9
Percentage of trials not receiving rewards	3.95%	1.64%	4.93%	2.96%	6.58%	2.96%
Mean percentage of trials not receiving rewards	2.79%±8.06%		3.94%±8.71%		4.76%±12.07%	
Mean (RT)	1862.31	1217.84	1774.57	1331.12	1869.42	1333.92
Standard deviation (SD)	68.73	108.92	125.53	117.55	74.47	86.20

Note: 304 trials in total on each n-back task (38 participants). Participants received feedback regarding the two criteria of accuracy of the n-back responses across 16 trials (8 trials for each 0-back and 2-back task condition) for each condition (monetary, humorous, and no reward).
